# Evaluation of the activity of a chemo-ablative, thermoresponsive hydrogel in a murine xenograft model of lung cancer

**DOI:** 10.1038/s41416-020-0904-9

**Published:** 2020-05-27

**Authors:** Seóna M. Rossi, Benedict K. Ryan, Helena M. Kelly

**Affiliations:** 1 School of Pharmacy and Biomolecular Sciences, Royal College of Surgeons in Ireland (RCSI), 123 St. Stephen’s Green, Dublin 2, Ireland; 2 Tissue Engineering Research Group, Department of Anatomy, Royal College of Surgeons in Ireland (RCSI), 123 St Stephen’s Green, Dublin 2, Ireland

**Keywords:** Drug development, Lung cancer

## Abstract

**Background:**

Minimally invasive intratumoural administration of thermoresponsive hydrogels, that transition from liquid to gel in response to temperature, has been proposed as a potential treatment modality for solid tumours. The aim of this study was to assess the inherent cytotoxicity of a poloxamer-based thermoresponsive hydrogel in a murine xenograft model of lung cancer.

**Methods:**

In vitro viability assessment was carried out in a lung cancer (A549) and non-cancerous (Balb/c 3T3 clone A31) cell line. Following intratumoural administration of saline or the thermoresponsive hydrogel to an A549 xenograft model in female Athymic Nude-Foxn1nu mice (*n* = 6/group), localisation was confirmed using IVIS imaging. Tumour volume was assessed using callipers measurements over 14 days. Blood serum was analysed for liver and kidney damage and ex vivo tissue samples were histologically assessed.

**Results:**

The thermoresponsive hydrogel demonstrated a dose-dependent cancer cell-specific toxicity in vitro and was retained in situ for at least 14 days in the xenograft model. Tumour volume increase was statistically significantly lower than saline treated control at day 14 (*n* = 6, *p* = 0.0001), with no associated damage of hepatic or renal tissue observed.

**Conclusions:**

Presented is a poloxamer-based thermoresponsive hydrogel, suitable for intratumoural administration and retention, which has demonstrated preliminary evidence of local tumour control, with minimal off-site toxicity.

## Background

Systemic intravenous chemotherapy has long been a central pillar of cancer treatment; however, the inherent physiological complexity of solid tumours presents a significant barrier to effective drug delivery and treatment. This results in a need for higher systemic doses of drug, leading to increased toxicity and patient morbidity, often with limited efficacy.^1^ More targeted approaches are required which allow for increased concentration of the active agent at the required site of action.^2,3^ In recent years, as the field of interventional oncology has grown, the potential for direct injection of chemotherapeutics into the tumour site using image-guided minimally invasive administration has increased.^4–6^ A challenge facing direct intratumoural instillation of chemotherapeutic solutions is the rapid clearance of these drugs from the tumour site, which results in inaccurate and unpredictable dosing, as well as toxicity to the surrounding healthy tissue.

Attention has turned to the development of delivery systems which can facilitate minimally invasive administration of therapeutics while ensuring retention and sustained release at the intended site of action. Polymeric drug eluting beads (DEB) are microparticles that are delivered during a transarterial chemoembolisation procedure, and have been proposed to improve sustained release of locally administered chemotherapeutics.^4^ Once delivered via catheter, the DEBs can act to block tumour-supplying blood vessels to cut off blood supply to the tumour mass, which will result in ischaemia and cell death, while simultaneously delivering a chemotherapeutic payload.^7,8^ Thermoresponsive hydrogels are another approach to locoregional tumour treatment. These hydrophilic polymers, exist as low viscosity liquids at room temperature, but undergo gelation in response to exposure at a characteristic temperature.^9^ This enables delivery directly to a solid tumour via minimally invasive procedures where upon transition to a gel, an in situ depot is formed.

**Fig. 1:**
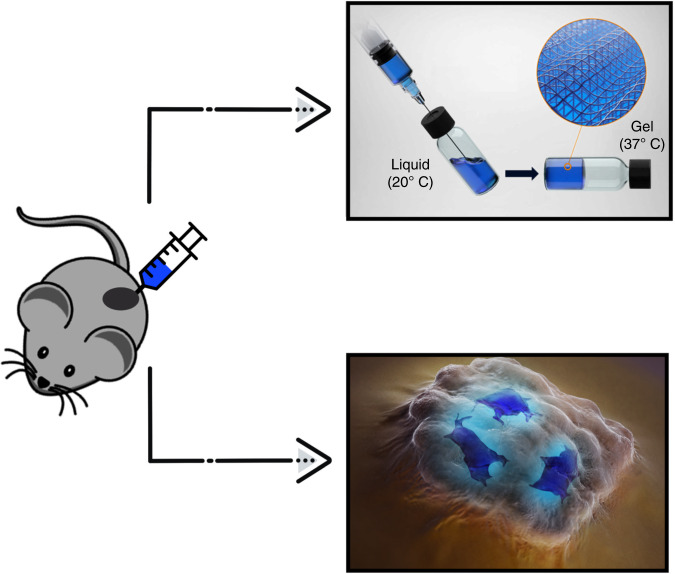
Schematic of intratumoural injection of a thermoresponsive hydrogel into a murine xenograft model of cancer. Thermoresponsive hydrogels are liquids at room temperature and undergo a characteristic phase transition to form a gel at body temperature. This facilitates the minimally invasive administration of a liquid via needle and the subsequent in situ gelation within the tumour.

The use of thermoresponsive hydrogels loaded with chemotherapeutic drugs for direct intratumoural administration in solid tumour treatment has been investigated in the pre-clinical and clinical setting (Fig. 1). Such an approach has been proposed as a safer and more effective method of treatment for patients with solid tumours than traditional systemic administration.^2,10–16^ Poloxamer-based thermoresponsive hydrogels, in particular, have been extensively evaluated in the pre-clinical setting with the superiority of this treatment modality compared to systemic administration established in a number of pre-clinical models of solid tumours.^17–20^ However, challenges relating to material properties, drug loading and dosing regimens, and overall efficacy have limited the clinical translation of this treatment approach for solid tumours.^16,21–23^

This paper investigates the use of a novel poloxamer-based thermoresponsive hydrogel, TGel, developed within our lab. Previous work carried out by our group demonstrated that this hydrogel could be formulated with suitable material characteristics for minimally invasive administration under image guidance.^24^ Here we assess the in vitro and in vivo biological properties of TGel, in a lung cancer model. TGel is shown to possess inherent chemo-ablative properties, negating the requirement for additional chemotherapy to be loaded into the formulation. Intratumoural administration of chemo-ablative TGel may present an opportunity to exert local tumour control and holds potential to act synergistically when used in combination with chemotherapy or other treatment modalities across a range of clinical indications from early stage to palliative treatment.

## Methods

Poloxamer 407 (P407) was obtained from BASF Corp. (Ludwigshafen, Germany), with proprietary modifications made to the final thermoresponsive hydrogel formulation (TGel). The A549 and Balb/c 3T3 clone A31 cell lines were purchased from LGC Standards (Middlesex, UK). An A549 cell line expressing firefly luc with puromycin resistance (SC043-luc) was purchased from AMS Biotechnology (Abingdon, UK). Dulbecco’s Modified Eagles Medium /Hams’ Nutrient Mixture F12 (DMEM:F12) (D8062), DMEM—high glucose (D6429), Penicillin 10,000 units/ml—Streptomycin 10 mg/ml solution (Pen/Strep), Trypsin, Aspartate Aminotransferase Activity Assay Kit (MAK055), Urea Assay Kit (MAK006) and neutral buffered formalin (NBF) 10% v/v were all purchased from Sigma Aldrich (MO, USA). Foetal bovine serum (FBS) and new-born calf serum (NBCS) were purchased from BioSera (Nuaillé, France). Cell Counting Kit-8 (CCK-8) was purchased from NBS Biologicals Ltd. (Cambridgeshire, UK). LIVE/DEAD® Viability/Cytotoxicity Kit for Mammalian Cells was purchased from Invitrogen Ireland. APC Annexin V Apoptosis Detection Kit with Propidium Iodide (PI) was purchased from BioLegend (CA, USA). Matrigel® Basement Membrane Matrix, Phenol Red-Free, *LDEV-Free (356237) was purchased from Corning® Life Sciences (MA, USA). XenoLight d-Luciferin - K+ Salt Bioluminescent Substrate (122799) was purchased from Perkin Elmer (MA, USA). The following controlled medicinal products required for animal anaesthesia and analgesia were used Ketamine (Narketan-10 100 mg/ml solution for injection, Vetoquinol UK Limited, UK), Xylazine (Chanazine 2% w/v solution for injection, Chanelle Pharmaceuticals Manufacturing Ltd., Ireland), Isoflurane (Isoflurin 100 mg/g Inhalation vapour, liquid, Vetpharma Animal Health, S.L., Spain).

### Cell culture

The human epithelial carcinoma cell line, A549 and a bioluminescent variant A549 cell line expressing firefly luc with puromycin resistance (A549-luc) were used for in vitro and in vivo studies respectively. Both A549 cell lines were maintained in vitro in 5% CO_2_, 90% humidity environment and cultured in DMEM:F12 supplemented with 10% v/v FBS and 1% v/v Pen/Strep solution. A murine fibroblast cell line, Balb/c 3T3 clone A31 was maintained in the same conditions and cultured using DMEM—high glucose supplemented with 10% v/v NBCS and 1% v/v Pen/Strep. Supplemented medium was replaced every three days, and cells were passaged when they had reached 80–90% confluency (A549 cells) or 60% confluency (Balb/c 3T3 clone A31 cells).

### In vitro studies

A549 cells were seeded at a density of 20,000 cells per well in a 24-well plate with 500 µL of supplemented medium. Cells were allowed to adhere for 24 h at 37 °C in a 5% CO_2_, 90% humidity environment. After 24 h, medium was removed from wells, and replaced with fresh supplemented medium. TGel was prepared according to proprietary specifications with routine quality control testing carried out for each batch to ensure appropriate thermoresponsive behaviour (Supplementary Fig. 1). Defined volumes of TGel (0, 10, 20 or 30 µL) were added to the fresh supplemented medium to bring the final volume of each well to 500 µL, based on methods by Ma et al.^10^ Plates were returned to the incubator at 37 °C in a 5% CO_2_, 90% humidity environment for 24 or 48 h. Following the pre-determined incubation period, plates were removed from the incubator, and the supernatant was discarded. Wells were washed once with Phosphate Buffered Saline and viability was assessed using a CCK-8 assay and Live/Dead staining.

CCK-8, a tetrazolium-based assay, which quantifies cellular viability based on the ability of the cell to produce dehydrogenase, was used in accordance with manufacturer’s instructions. In brief, 200 µL fresh supplemented medium was added to each well. In all, 20 µL of CCK-8 reagent was added to each well, and the plates were returned to the incubator for 90 min. The same volume of CCK-8 in medium was also added to wells with no cells seeded to act as a control for absorbance detected from cell free medium (blank). In all, 100 µL of the CCK-8 incubated medium from each well was then transferred to a 96-well plate and absorbance was read at 450 nm on a Varioskan Flash Plate Reader (Thermo Fisher Scientific, MA, USA). Medium treated cells were taken as 100% viability, and the viability of each treatment group was expressed as a percentage of this (Eq. 1).

1 $$\frac{{{\mathrm{Ave.}}\;{\mathrm{abs.}}\;{\mathrm{of}}\;{\mathrm{treatment}}\;{\mathrm{group}} - {\mathrm{Ave.}}\;{\mathrm{abs.}}\;{\mathrm{of}}\;{\mathrm{blank}}}}{{{\mathrm{Ave.}}\;{\mathrm{abs.}}\;{\mathrm{of}}\;{\mathrm{Medium}}\;{\mathrm{group}} - {\mathrm{Ave.}}\;{\mathrm{abs.}}\;{\mathrm{of}}\;{\mathrm{blank}}}}\times\frac{{100}}{1}$$

All experiments were conducted in quadruplicate, and data shown is representative of the mean of three independent experiments + SEM.

Live/Dead staining was performed as a qualitative indicator of viability and cytotoxicity of treatment; Live cells were stained green using Calcein AM and dead cells were stained red using Ethidium homodimer-1. Cell staining was carried out according to the manufacturers protocol.^25^ In all, 2.5 µL Calcein AM and 10 µL Ethidium homodimer-1 was added to 5 ml PBS. 300 µL of the Calcein AM/Ethidium homodimer-1 solution was added to each treatment well and allowed to develop for 30 min. Stains were then removed, and 300 µL of PBS was added to the wells. Live and dead cells were fluorescently imaged individually using blue (FITC/GFP) and green (TRITC) filters respectively on the same field of view with a Leica DMIL microscope (Leica Microsystems, Switzerland). Image J was used to generate composite images of cell viability/cytotoxicity.

### Apoptosis analysis

Analysis of A549 cell apoptosis after treatment with 30 µL of supplemented medium or TGel was evaluated using APC Annexin V Apoptosis Detection Kit with PI according to manufacturer’s instructions, and detected via flow cytometry.^26^ Cells were seeded at 200,000 cells per well in 2 mL supplemented medium in six-well plates (Corning™ Costar™, NY, USA), and allowed to adhere for 24 h. Medium was removed, and 30 µL of fresh supplement medium or TGel was added to each well with 1970 µL of fresh supplemented medium. Plates were returned to the incubator for 24 h. After 24 h treatment plates were washed once with PBS. Cells were detached from the wells via trypsinisation and centrifuged at 1200 rpm for 5 min to produce a cell pellet. Supernatant was removed from the cell pellet, and cells were then washed twice with FACS buffer. Cells were resuspended in 100 µL Annexin V binding buffer in a 5 ml tube. 5 µL of APC Annexin V and 10 µL of Propidium Iodide solution were added to each tube. Tubes were gently vortexed and incubated for 15 min at room temperature (< 25 °C) in the dark. 400 µL of Annexin V Binding Buffer was added to each tube, and cells were analysed by flow cytometry (BD Canto, BD Biosciences, CA, USA).

All experiments were performed in triplicate, and data shown is representative of the mean of two independent experiments + SEM.

### Animals

All animal experiments were approved by the Animal Research Ethics Committee, Royal College of Surgeons in Ireland (REC no. 1389) and by the national scientific animal regulatory authority, the Health Products Regulatory Authority (HPRA), and were conducted in accordance with European Union legislation (Directive 2010/63/EU).

Female Hsd:Athymic Nude-Foxn1nu mice (20–25 g weight) were purchased from Envigo (Huntingdon, UK), and housed under specific pathogen free (SPF) conditions with controlled temperature of between 20–24 °C, humidity between 45–65%, and 12 h light/dark cycle. All animal handling was conducted in a dedicated SPF facility under laminar airflow using aseptic procedures. All mice were group housed (*n* = 4 per cage) and maintained on autoclaved water and food available ad libitum. Regular welfare checks were conducted on all mice as per project authorisation, including body weight measurements, and physical and behavioural observations.

All procedures detailed below were carried out under anaesthesia, outside the home cage. Inhalation-based anaesthesia was induced using 4% v/v isoflurane and oxygen in an induction chamber, with 2% v/v isoflurane used as maintenance anaesthesia in induction chamber or nose cone. If a systemic anaesthetic regimen was required, ketamine (90 mg/kg) and xylazine (10 mg/kg) were administered via the intra-peritoneal route. In all cases, absence of pedal withdrawal reflex was confirmed prior to commencement of the procedure to ensure deep anaesthesia was achieved. Following completion of procedures, mice were placed individually in a recovery cage adjacent to a heating lamp until sedation was removed, at which point they were returned to the home cage.

### In vivo studies

A549-luc cells were suspended in a 1:1 PBS:Matrigel mixture at a density of 1 × 10^7^ cells/ml, after being trypsinised and kept on ice until use.^27^ Following confirmation of successful inhalation anaesthesia, 100 µL of cell suspension (1 × 10^6^ cells) was injected via subcutaneous injection into the flank of the mouse in the lower right-hand quadrant, using a 29G syringe. The needle was left in place for 30 s after injection, rotated and removed slowly to prevent leakage of cell suspension from injection site.

Once tumours were palpable, dimensions of the tumour were taken externally using digital callipers to measure the length (l) and width (w). Tumour volume was derived using Eq. 2.^28^

2 $$\frac{{{\mathrm{length}}\;\times\;{\mathrm{width}}^2}}{2}$$

Intratumoural administration of the sterilised TGel or saline (*n* = 6 per group) was performed under inhalation anaesthesia once tumour volume had reached 250 mm^3^ ± 50 mm^3^. In all, 100 µL of TGel formulation or saline (acting as a control) for injection was loaded into a 1 ml syringe with 22G needle and kept on ice prior to administration.

Tumours were stabilised using forceps and secured from beneath to minimise risk of needle piercing through tumour. The needle was inserted into the tumour at the base of the tumour, advanced until the tip was completely submerged. The final location of the tip was approximated to the mid-point of the tumour and the entire volume of required formulation was expelled slowly. The needle remained in place for 30 s following completion of injection to allow for gelation of the thermoresponsive hydrogel to occur, rotated and removed slowly to prevent backflow of injected material.

Post-intratumoural administration of TGel or control, tumour volume was measured at 2 h, Day 2, Day 7, Day 9 and Day 14 to monitor disease progression.

In vivo imaging of both tumours and the thermoresponsive hydrogel was conducted using an IVIS® Spectrum In Vivo Imaging System (Perkin Elmer, MA, USA) at Day 0 and 14. Visualisation of the bioluminescent A549-luc xenograft was achieved following intraperitoneal injection of freshly prepared D-Luciferin solution in PBS (150 mg/kg). Images were acquired between 10 and 12 min post-D-Luciferin administration as determined by kinetic curve, under inhalation anaesthetic. The thermoresponsive hydrogel formulation included a fluorescent tag to allow for qualitative in vivo imaging to confirm localisation of material.^29^

Animals were killed at Day 14, following administration of systemic anaesthesia as outlined above. Under deep anaesthesia, terminal cardiac puncture was performed, using a 1 ml luer-slip syringe (B Braun, Melsungen, Germany) and 21G needle. Following terminal cardiac puncture, death of the animal was confirmed using cervical dislocation. In all, 300 µL of blood was collected in a K_3_EDTA anti-coagulation tube (Microvette 500 K3E, Sarstedt, Nümbrecht, Germany) and analysed immediately for white blood cell count using a Sysmex KX-21N haematology analyser (Sysmex Corp., Kobe, Japan).

The remaining blood sample was collected in a 2 ml Eppendorf allowed to stand for ~30 min to coagulate. Samples were then centrifuged at 4700 rpm for 5 min. Serum was carefully removed from the centrifuged tube and frozen at –80 °C until analysis. Serum was analysed using Aspartate Aminotransferase (AST) and Urea assay kits according to manufacturer’s instructions. Absorbance was read at 450 nm and 570 nm, respectively, using a Victor^2^ 1420 plate reader (Perkin Elmer, MA, USA).

### Collection of tissue samples and histology

Liver and kidneys were identified and excised. The tumour was then removed from the lower right-hand quadrant of the flank. Excess tissue and fascia were carefully removed from excised organs and tumour. Visual images of the excised tumour were taken, and fluorescent imaging of the excised tumour, liver and kidney was carried out immediately following excision.

Histological tissue processing and haematoxylin and eosin (H&E) stained and unstained slides production was performed by Novaxia Laboratories (Saint-Laurent-Nouan, France). Kidney and liver tissues (*n* = 6 per group of each organ) collected at necropsy were placed in 10% v/v neutral buffered formalin fixative solution for 24 h and subsequently dehydrated in increasing concentrations of ethanol until storage in 70% v/v ethanol solution. Formalin-fixed tissues were trimmed, processed and embedded into paraffin blocks at Novaxia’s facilities. Representative 5 µm thick sections from each individual paraffin-embedded kidney and liver samples were prepared and stained with H&E. Whole slide digital scans were produced by the Nanozoomer (Hamamatsu, Japan) at 20X magnification at Biodoxis laboratory facilities.

### Statistical analysis

For in vivo studies the sample size calculations were informed by a literature review and based on an expected tumour volume of 500 mm^3^ ± 85 mm^3^ (mean ± SD) at 2 weeks post intratumoural administration in control (saline) treated animals. A volume change of ~35% was pre-determined as clinically significant. A group size of 6 was calculated to be necessary to detect a change in tumour volume of 35% with an alpha value of 0.05 and power (β) of 0.8. (Actual Power: 0.87). Two-way analysis of variance (ANOVA), with Tukey's multiple comparisons test, was conducted to determine statistically significant difference in in vitro viability post exposure to medium or TGel in A549 and Balb/c 3T3 clone A31 cell lines. Statistically significant differences in apoptotic measures and blood chemistry were determined using an unpaired t test between saline and TGel treated groups. Two-way ANOVA with repeated measures, with Sidak’s multiple comparisons test, was used to determine statistically significant differences between tumour volume measurements made. All statistical tests were performed using GraphPad Prism v6 (GraphPad Software Inc., CA, USA). Error is reported as SEM and statistical significance was determined using a probability value of *p* < 0.05.

## Results

**Fig. 2:**
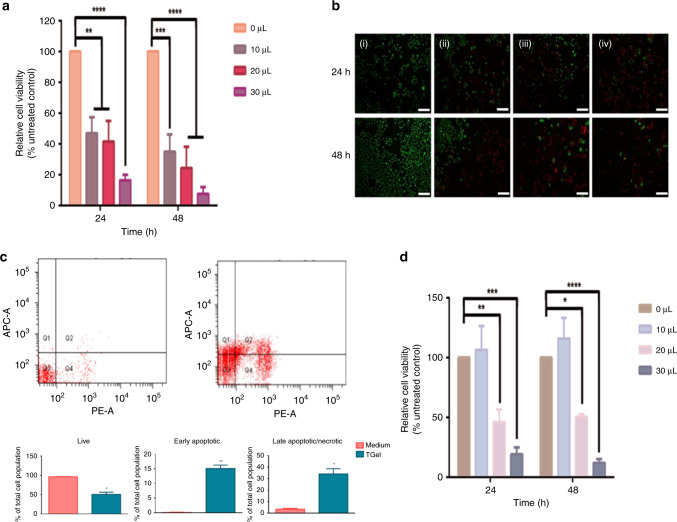
Treatment with TGel significantly reduced viability of A549 cells. **a** Relative viability of A549 cells treated with 0–30 µL of TGel for 24 and 48 h (*n* = 3). Significance was determined using a two-way ANOVA, with Tukey's multiple comparisons test. ** = *p* < 0.01, *** = *p* < 0.001, **** = *p* < 0.0001. **b** Representative images of Live/Dead staining of A549 cells at 24 and 48 h with 0,10, 20 and 30 µL of TGel (i–iv, respectively). Live cells stained green, dead cells stained red. Magnification, ×10. Scale bar, 200 µm. **c** Flow cytometric analysis of apoptosis of Medium (left, top) and TGel (right, top) treated A549 cells for 24 h in vitro. Proportion of live, early apoptotic, late apoptotic/necrotic cells treated with Medium or TGel (bottom). Q 1 = early apoptotic cells, Q 2 and Q 4 = late apoptotic/necrotic cells and Q 3 = live cells (*n* = 2). Significance within treatment groups was determined using an unpaired *t* test. * = *p* < *0.05,* ** = *p* < 0.01. **d** Relative viability of Balb/c 3T3 clone A31 cells treated with TGel for 24 and 48 h (*n* = 3). Significance was determined using a two-way ANOVA, with Tukey's multiple comparisons test. * = *p* < 0.05, ** = *p* < 0.01, *** = *p* < 0.001, **** = *p* < 0.0001.

All doses of TGel evaluated demonstrated a statistically significant decrease in the viability of A549 cells at 24 and 48 h (Fig. 2a), with viability data qualitatively confirmed using Live/Dead staining (Fig. 2b). The reduction in cell viability was seen to occur in a dose-dependent manner (10 µL: 34.92% ± 11.18% [*p* = 0.0005], 30 µL 7.51% ± 4.47% [*p* = <0.0001] at 48 h).

Apoptosis analysis of TGel treated A549 cells in vitro revealed a statistically significantly reduced viability of A549 cells compared to Medium alone (Fig. 2c). Treatment with TGel reduced the percentage of live cells to 50.85% ± 5.75% (*p* = 0.0157), with the greatest number of dead cells in the late apoptotic/necrotic stages (34.05% ± 4.55%, *p* = 0.022).

Treatment of a non-cancerous cell line, Balb/c 3T3 clone A31 with 0–30 µL of TGel was then used to assess the cytotoxicity in off-target cells (Fig. 2d). Treatment with 10 µL of TGel did not negatively impact the viability of the Balb/c 3T3 clone A31 cells at 24 and 48 h (106.44% ± 19.89% and 115.89% ± 17.22% respectively). Increasing TGel dose to 20 µL and 30 µL resulted in a statistically significant decrease in viability compared to untreated cells (20 µL: 50.61% ± 2.36% [*p* = 0.0178] and 30 µL: 11.77% ± 3.45% [*p* = <0.0001] at 48 h).

**Fig. 3:**
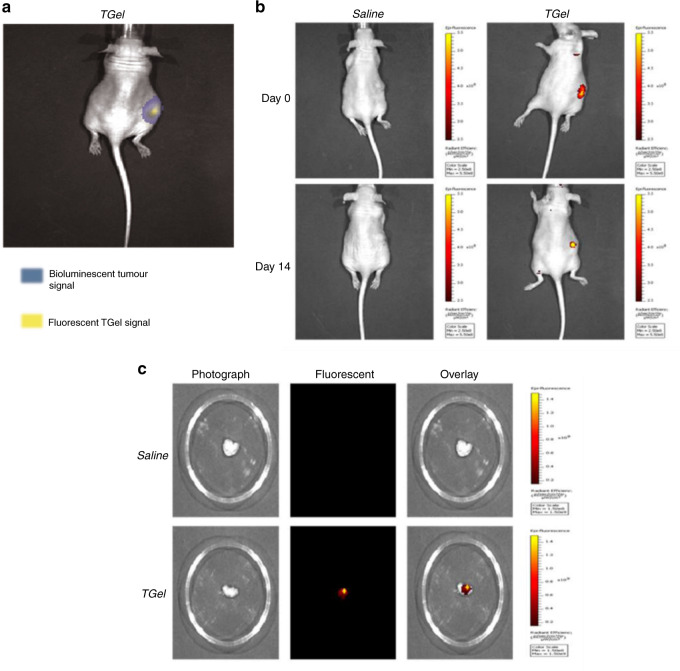
TGel was retained at site of intratumoural injection for 14 days in vivo. **a** Representative overlay images of bioluminescent A549-luc cells post intratumoural administration of 100 µL of fluorescently tagged TGel at Day 0. Blue represents bioluminescent signal from A549-luc cells, Yellow represents fluorescent signal from TGel. **b** Representative fluorescent images at Day 0 (top) and Day 14 (bottom) post intratumoural administration of saline (left) or TGel (right). **c** Representative images (greyscale photograph [left], fluorescent signal [centre] and overlay [right]) of saline (top) or TGel (bottom) treated tumours excised on Day 14 post intratumoural administration.

In vivo localisation of TGel was confirmed 2 h after intratumoural injection by creating an overlay image of the bioluminescent signal from the A549-luc cells and the fluorescent signal from the fluorescently tagged formulation (Fig. 3a). Intratumoural localisation was considered successful if the fluorescent signal was in the region of the bioluminescent signal. All injected formulations imaged at 2 h post-intratumoural administration qualitatively indicated localisation of the gel at the tumour site, with in vivo imaging indicating retention at Day 14 also (Fig. 3b). Ex vivo assessment showed TGel to be still present in all treated tumours at Day 14 (Fig. 3c).

**Fig. 4:**
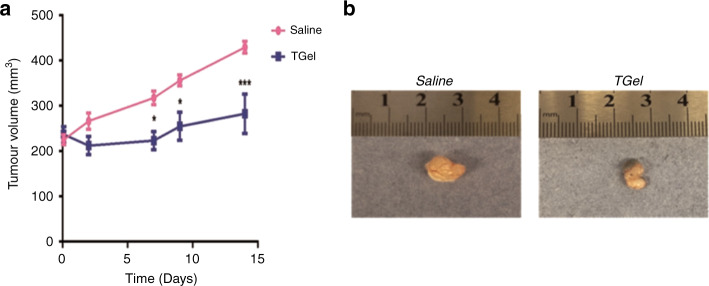
Intratumoural administration of TGel significantly reduced tumour volume increase. **a** Tumour volume (mm^3^) following intratumoural administration of 100 µL of saline or TGel over 14 days. Data shown is represented as the mean ± SEM (*n* = 6 mice per group). Significance was determined using a repeated measures two-way ANOVA, with Sidak’s multiple comparisons test. * = *p* < 0.05, *** < *p* = 0.001 compared to volume of saline treated tumours at the same timepoint. **b** Representative images of excised tumours at Day 14 following intratumoural administration of Saline (left) or TGel (right).

Treatment with TGel significantly reduced tumour volume increase compared to saline treated control 14 days after intratumoural administration (*p* = <0.0001) (Fig. 4a). The final volume of saline treated tumours was recorded as 429.34 mm^3^ ± 12.87 mm^3^ and the final tumour volume of TGel treated tumours was 282.52 mm^3^ ± 43.02 mm^3^. Macroscopic inspection of excised tumours at Day 14, visually supported the quantitative results (Fig. 4b).

**Fig. 5:**
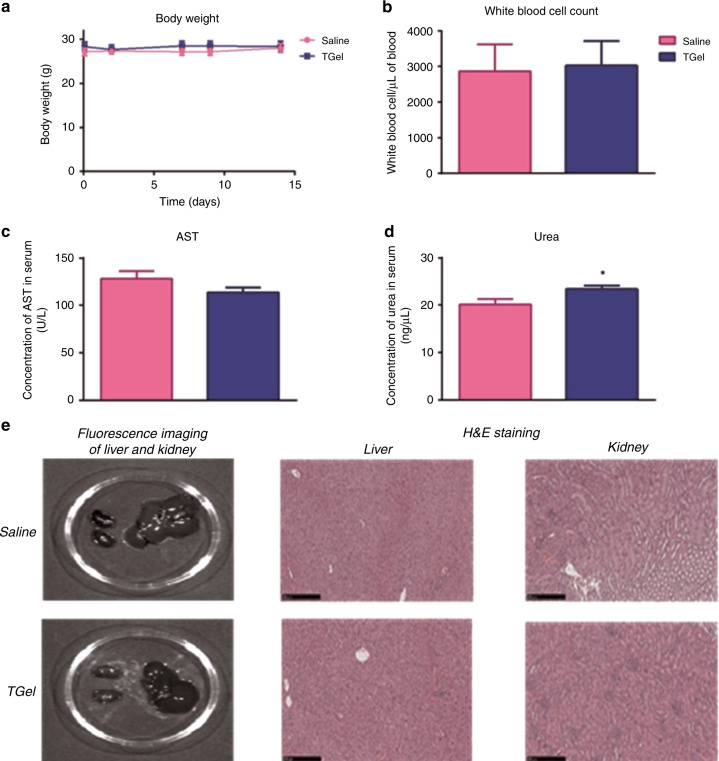
Intratumoural administration of TGel did not result in widespread off-site toxicity. Intratumoural administration of saline or TGel did not cause severe acute off-site toxicity for up to 14 days with no significant alterations in **a** body weight, **b** white blood cell count or **c** blood serum levels of Aspartate transaminase (AST). **d** Urea levels were statistically significantly higher in TGel treated mice when compared to saline at 14 days post intratumoural administration. **e** Representative fluorescent images of kidneys and liver excised at Day 14 from mice treated with intratumoural administration of saline (left, top) or TGel (left, bottom). Representative H&E staining of excised liver (centre) and kidney (right) tissue day 14 post intratumoural administration of saline (top) or TGel (bottom). Data shown is represented as the mean + SEM (*n* = 6). Significance was determined using an unpaired *t* test for (**b**–**d**). ns = *p* > 0.05, * = *p* < 0.05.

Throughout the 14-day study period no physical or behavioural changes were observed in the treatment group, with 100% survival in both groups recorded at Day 14. No statistically significant fluctuations in body weight, white blood cell count or AST levels were observed during the 14-day study (Fig. 5a–c). A statistically significant difference in urea levels was noted between the treatment and control group at Day 14 post intratumoural administration (*p* = 0.04) (Fig. 5d). Fluorescent imaging of excised liver and kidney indicated there was no detectable levels of TGel present in these organs after treatment (Fig. 5e, left bottom). H&E staining demonstrated no structural damage to organs with minimal and focal mixed or mononuclear cell interstitial inflammation observed in two liver sections (Fig. 5e, centre top and bottom). These lesions were considered incidental in origin and not treatment-related. No lesions were observed in any of the kidney sections examined (Fig. 5e, right top and bottom).

## Discussion

The results presented in this study provide preliminary evidence to support the use of a novel poloxamer-based thermoresponsive hydrogel formulation in the locoregional treatment of solid tumours. In addition to demonstrating retention for at least 14 days at the tumour site, TGel also shows significant inherent chemo-ablative properties with minimal off-site toxicity in a preliminary proof of concept murine xenograft model of lung cancer. Lung cancer was chosen for proof of concept due to the potential for intratumoural applications in a variety of clinical settings including both early stage single and palliative scenarios, coupled with the relative ease of access for intratumoural delivery.^30–32^

Dose related cytotoxicity of TGel was observed in vitro, however of interest was the increased cytotoxicity observed in the A549 lung cancer cell line relative to the Balb/c 3T3 cells, which are commonly used for cytotoxicity testing,^33^ indicating some degree of cancer cell specificity. At the lowest concentration used, TGel was seen to have no effects on the Balb/c 3T3 cells, whereas the same dose administered to A549 cells resulted in a statistically significant decrease in viability. Apoptosis analysis of TGel treated A549 cells revealed that the majority of cell death induced by the chemo-ablative formulation was found to be in the late apoptotic or necrotic stage after 24 h of treatment, further supporting its cytotoxic efficacy in cancer cells.^10^ Based on these preliminary in vitro results, the formulation was progressed to in vivo testing in an A549 murine xenograft model.

In vivo fluorescence imaging revealed that TGel was successfully localised at site of injection and retained at the tumour site for the duration of the 14-day study. This was confirmed by ex vivo fluorescence imaging of excised tumours. Retention at the tumour site is of significant clinical importance in optimising efficacy of intratumoural administration and reducing localised toxicity as it enables more sustained exposure at the required site of action. The poor mechanical strength associated with poloxamer hydrogels has been previously reported to result in rapid in vivo disintegration.^34,35^ However proprietary modifications made to this formulation have enabled an extended disintegration profile.^24^

Over the course of the 14-day study TGel was shown to significantly reduce tumour volume increase relative to saline, confirming in vitro data. This efficacy is due to two potential mechanisms of action, with a combination of both most likely contributing to the overall activity observed.

Firstly, it is possible that the hydrogel is exerting an embolic effect, in which it prevents blood flow to the tumour, thereby inducing cell death. Embolisation, both alone and in combination with loco-regional chemotherapy delivery, is currently used as a first line treatment approach in certain types of hepatocellular carcinoma (HCC). Clinical efficacy is via dual mechanism of action with the embolic agent cutting off blood supply to the tumour mass, resulting in ischaemia and cell death, while the chemotherapy agent exerts a pharmacological effect.^36–38^ The liver is well vascularised, and as such, other blood vessels can maintain adequate blood supply to the rest of the organ. This has been recognised as a limiting factor in the translation of this technique to other tumours, since a well vascularised organ is required to ensure that healthy tissue is not compromised. As TGel delivers directly into the tumour, this may provide potential for broader clinical applications in organs which are not as well vascularised as the liver.

Secondly, poloxamer polymers have significant documented evidence to support a cancer cell directed mechanism of action, with multiple pharmacological target(s) of this including fluidisation of the cellular membrane, ATP depletion, inhibition of drug efflux and reduction in GSH/GST detoxification activity.^39–42^ The prolonged retention of TGel at the tumour site enabled sustained exposure to poloxamer at the tumour site. It is hypothesised that this prolonged retention facilitates both physical and chemical effects at the tumour site resulting in the chemo-ablative effect observed with tumour growth inhibition over the 14-day study period.

Pre-clinical assessments of intratumoural administered thermoresponsive hydrogels generally focus specifically on their use for drug delivery, rather than their inherent cytotoxicity. However, formulations with inherent chemo-ablative properties have been reported in the literature as drug-free methods of cancer treatment. Indeed, one of the earliest examples of loco-regional solid tumour treatment was the use of ethanol ablation in HCC, involving the direct injection of 70% ethanol into the tumour under image guidance to track distribution. It has been employed in the local treatment of small HCC tumours (<3 cm) since the 1980s,^43^ although its use has reduced in recent times as more effective approaches were developed. Building on this approach, an ethanol based hydrogel has also been explored as a chemo-ablative treatment approach for solid tumours.^44^

Preliminary indicators including body weight, white blood cell count and liver markers showed no evidence of off-site toxicity. The low level of toxicity of poloxamer formulations to healthy cells is a commonly cited benefit to their use in a clinical setting, with a number of commercially approved formulations available.^45,46^ The slower rate of disintegration of TGel contributes to the minimal side-effect profile observed, as TGel is broken down slowly and excreted from the body over time. The elevation in urea may be explained by the fact that poloxamer is excreted through the kidneys.^47^ No structural damage to the renal tissue was observed in the treated mice, indicating that further studies are required to determine whether elevated urea levels are a transient effect or indicative of tissue damage.

These results confirm the feasibility for intratumoural administration and retention of the thermoresponsive hydrogel and indicate that it exerts localised chemo-ablative properties. It is acknowledged that heterotopic, xenograft models are not wholly representative of the clinical reality, and due consideration should be given to inter-species variation, when interpreting the clinical significance of these results. Establishment of these initial treatment parameters will enable design and execution of further in vivo studies to investigate in more detail the chemo-ablative mechanism of action, dosing and administration regimens and toxicity. This chemo-ablative, thermoresponsive hydrogel may offer a unique alternative to systemic chemotherapeutic treatment to effect local tumour control.

## Supplementary information

Supplemental material_Figure 1

## Data Availability

Data sharing not applicable to this article as no datasets were generated or analysed.
