# Supplementary material for: Evaluation of the activity of a chemo-ablative, thermoresponsive hydrogel in a murine xenograft model of lung cancer
Source: Br J Cancer. 2020 May 27;123(3):369–77. doi: 10.1038/s41416-020-0904-9 (PMC7403591; doi:10.1038/s41416-020-0904-9)
Supplement: Supplementary file 1 — Supplemental material_Figure 1 [file 41416_2020_904_MOESM1_ESM.pdf]

Supplementary information

Oscillatory temperature sweeps from 20°C – 40°C (cone/plate geometry, 40 mm diameter, 4° angle, AR-1000 constant stress rheometer, TA instruments, DE, USA) were carried out on all formulations used to ensure appropriate sol-gel transition temperature (29°C). Temperature was increased at a rate of 1°C/min, with constant oscillatory shear stress (5 Pa) and angular frequency (1 Hz). All samples were analysed in triplicate.

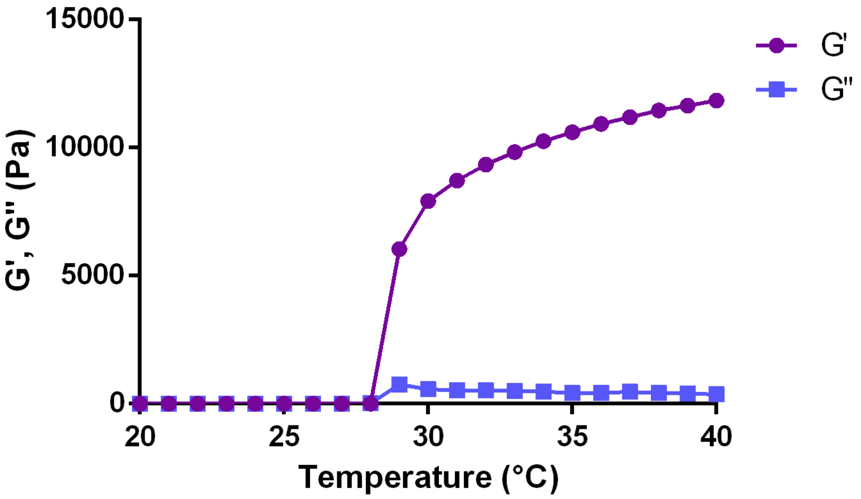

Supplementary Figure 1. *Rheogram of oscillatory temperature sweeps from 20ºC - 40ºC of TGel. Data shown is representative of the norm.*
